# Gate-Tunable Spin Transport and Giant Electroresistance in Ferromagnetic Graphene Vertical Heterostructures

**DOI:** 10.1038/srep25253

**Published:** 2016-04-29

**Authors:** Nojoon Myoung, Hee Chul Park, Seung Joo Lee

**Affiliations:** 1 Department of Material Science and Engineering, University of Ioannina, Ioannina 45110, Greece; 2 Center for Theoretical Physics of Complex Systems, Institute for Basic Science, Daejeon 34051, Republic of Korea; 3 Quantum-functional Semiconductor Research Center, Dongguk University, Seoul 100-715, Republic of Korea

## Abstract

Controlling tunneling properties through graphene vertical heterostructures provides advantages in achieving large conductance modulation which has been known as limitation in lateral graphene device structures. Despite of intensive research on graphene vertical heterosturctures for recent years, the potential of spintronics based on graphene vertical heterostructures remains relatively unexplored. Here, we present an analytical device model for graphene-based spintronics by using ferromagnetic graphene in vertical heterostructures. We consider a normal or ferroelectric insulator as a tunneling layer. The device concept yields a way of controlling spin transport through the vertical heterostructures, resulting in gate-tunable spin-switching phenomena. Also, we revealed that a ‘giant’ resistance emerges through a ferroelectric insulating layer owing to the anti-parallel configuration of ferromagnetic graphene layers by means of electric fields via gate and bias voltages. Our findings discover the prospect of manipulating the spin transport properties in vertical heterostructures without use of magnetic fields.

Graphene, a honeycomb-like single layer crystal of carbon atoms, has been attracting a lot of attention in the recent decade both in terms of fundamental interests and technology. Amongst the various aspects of graphene, one of the most promising potentials is that it has extraordinary transport properties such as high carrier mobility and long mean free path^1^,^2^,^3^. Despite these advantages for high-speed device applications, the use of single layer graphene for practical nanoelectronic devices, like field-effect transistors (FETs), is limited because of the low current on/off ratio^4^,^5^,^6^ that implies how effectively it generates digital signals. This limitation mainly stems from the intriguing relativistic transport phenomena in graphene, so-called Klein tunneling, which results in massless and chiral Dirac fermions that can perfectly pass through electrostatic potential barriers^7^,^8^,^9^.

Recently, an alternative platform has emerged for graphene FETs ,where graphene and other two dimensional layers are stacked vertically^10^,^11^. For graphene–hexagonal boron nitride (hBN)–graphene vertical heterostructures, the vertical current density can be largely modulated by controlling quantum tunneling through an atomically thin hBN layer via gate voltage^10^,^12^. Larger current on/off ratios can be achieved by using small-bandgap layered materials as a tunneling insulator^10^,^13^. Owing to the huge variety of structures and properties in vertical heterostructures of 2D materials, many promising and interesting research topics have been considered, e.g., field-effect transistors^10^,^14^, resonant tunnel diodes^15^,^16^, and photodetectors^17^,^18^. In particular, the vertical heterostructure platform can also be a good candidate for graphene-based spintronics^13^,^19^,^20^. The long spin-coherent length of graphene^22^,^23^,^24^,^25^ allows for the fabrication of spintronic devices using graphene sheets as spin transport channels, once the tunneling current is well spin-polarized through the vertical heterostructures.

In this article, we investigate the spin-resolved transport through the vertical heterostructures with ferromagnetic graphene (FMG). We show that the control of the spin transport through the structure can be achieved by electrically manipulating the spin configurations in FMG sheets. The spin-resolved band structure is taken into account to describe the electronic states of FMG, and the spin-resolved tunneling current density is calculated for two different combinations of heterostructures: FMG–normal insulator(NI)–FMG and FMG–ferroelectric insulator (FEI)–FMG. We also show that the giant electroresistance emerges for the anti-parallel configuration of FMGs when the sandwiched insulator is replaced by an FEI.

The system studied in this article is a vertically stacked heterostructure which is formed by FMG and an insulating layer [see Fig. 1a]. The sandwiched insulator and the graphene sheets play roles of a tunnel barrier and conducting channels, respectively. Dual-gated device structures are considered to control the same amount of the carrier densities on both graphene sheets^26^. The proximity interaction between a ferromagnetic insulator such as europium oxide (EuO) and graphene is able to induce ferromagnetism in graphene^27^,^28^,^29^,^30^. The spin-splitting in FMGs is responsible for spin transport in laterally formed FMG heterojunctions^31^,^32^. The bias voltage *V*_*b*_ can be applied between two graphene sheets, yelding the tunneling current through the insulating layer. The electronic properties of FMG are characterized by its spin-resolved electronic states^28^:


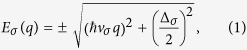


where *σ* = ±1 for spin-up and down states of Dirac fermions, *v*_↑_ = 1.15 × *v*_*F*_ and *v*_↓_ = 1.4 × *v*_*F*_ are Fermi velocities for each spin with *v*_*F*_ = 10^6^ m/s, Δ_↑_ = 134 meV and Δ_↓_ = 98 meV are the spin-resolved bandgaps, as displayed in Fig. 1(b). Here, we assume that the Fermi level of FMG is set in the mid-gap. For the proximity-induced ferromagnetic graphene, the valley degeneracy of the pristine graphene has been broken by interactions between carbon and europium atoms^28^. Particularly, depending on the position of the Fermi level, the FMG can be fully spin-polarized–at positive or negative unity–by adjusting the gate voltage via both gate electrodes (see Supplementary Information).

As a starting point, we introduce our vertical transport model used in this study. We consider the elastic tunneling of Dirac fermions in terms of energy, and scattering effects are taken into account by applying the current density formula. Also, we assume that the magnetizations of two FMG layers are parallel in the absence of external magnetic fields. The spin-resolved vertical tunnelig current is formulated with the interlayer transition matrix element based on WKB approximation^26^,


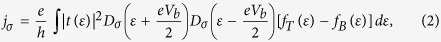


where 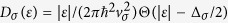 is the spin-resolved density of states (SDOS) with the spin-resolved Fermi velocities (see Fig. 1c), and 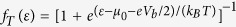 and 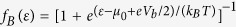 are the Fermi-Dirac distributions on top and bottom FMG layers, respectively. The interlayer transition matrix element is given by


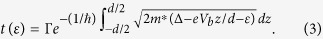


Here, m^*^ is the effective mass of the tunnel barrier material, Δ is the barrier height of the tunneling insulator, *V*_*b*_ is bias voltage which is applied via two graphene sheets, and *d* is the thickness of the tunneling insulator. Note that Γ is an energy-independent prefactor which represents the momentum scattering of Dirac fermions by disorders such as defects or phonons inside the tunnel barrier material. In other words, Γ = 1 means no scattering mechanism while Dirac fermions tunnel through the tunnel barrier, and on the other hand, the smaller Γs indicate more diffusive vertical transport through the tunnel barrier.

Carrier density on graphene layers is controlled by field-effects via gate electrodes. In the absence of bias voltage, the chemical potentials on both graphene layers are in equilibrium, leading to no net tunneling current density. The dual-gated platform is considered to fix and maintain the same carrier densities in the top and the bottom gate electrodes^26^, resulting in a symmetric gated structure, i.e., *V*_*TG*_ = *V*_*BG*_ ≡ *V*_*G*_. This assumption allows us to simplify the problem with fixed chemical potentials on both graphene layers in equilibrium, i.e., 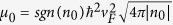. By using the electrostatic capacitor model, *n*_0_ is proportional to the gate voltage *V*_*G*_, i.e., *n*_0_ = *αV*_*G*_ where *α* is the proportional constant depending on the substrate (superstrate) materials between a graphene layer and the bottom (top) gate electrode. When bias voltage is applied to both graphene layers, their chemical potentials are shifted and equilibrium is broken, resulting in non-zero tunneling current denslty throughout the vertical heterostructure.

## Results

### Spin transport through FMG-NI-FMG heterostructures

Figure 2 shows spin-resolved vertical transport through the FMG-NI-FMG heterostructure. In the present study, Δ = 1.5 eV and m^*^ = 0.5 *m*_*el*_ with the bare mass of an electron *m*_*el*_ are used for the calculations, which are approximately compatible with typical 2d materials such as MoS_2_, WS_2_, etc.^33^,^34^,^35^,^36^. In the same context, the thickness of the NI layer is taken as 1 nm which is compatible with few-layer 2d material cases^14^. The spin transport is characterized by the spin-polarization of the tunneling current density,


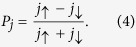


**Figure 1:**
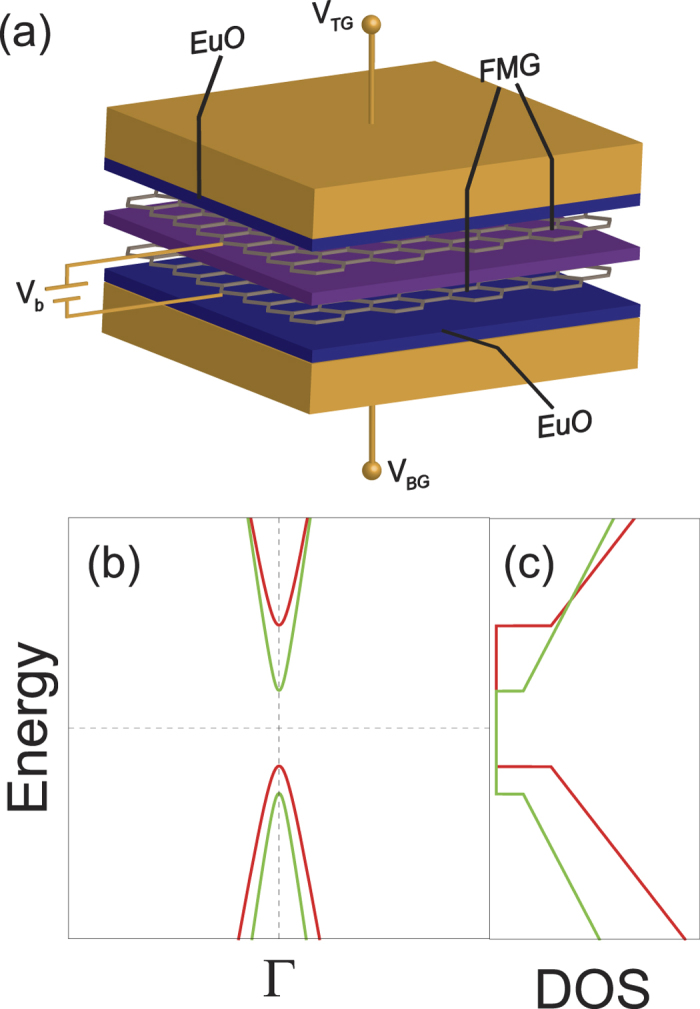
Model of heterostructure and electronic properties of ferromagnetic graphene (FMG). (**a**) Schematics of the vertical heterostructures with FMG and a tunneling insulator. (**b**) Spin-resolved band structures and (**c**) spin density of states (SDOS) of FMG. Red and Green solid lines represent spin-up and down states in FMG with spin-resolved bandgaps and Fermi velocities.

**Figure 2:**
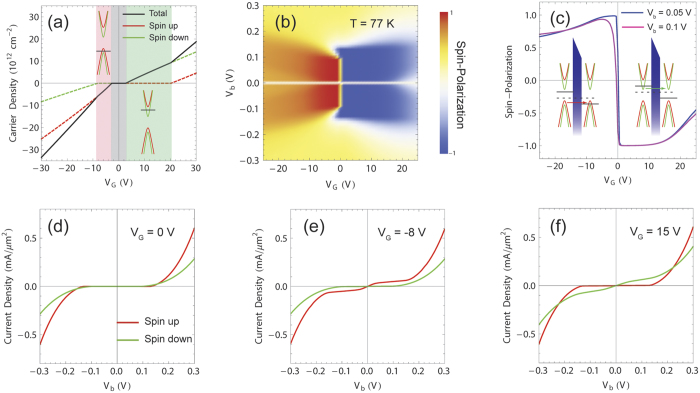
Spin transport through an FMG-NI-FMG hetrostructure. (**a**) Spin-resolved carrier densities and the corresponding total carrier density on a FMG layer versus gate voltage, in the absence of bias voltage. Left and right shaded regions represent pure spin-polarization, which are denoted as inset diagram. Middle shaded region corresponds to the forbidden zone where no Dirac fermions are allowed. (**b**) Color map of the spin-polarization of the tunneling current density as functions of bias and gate voltages. For *V*_*b*_ = 0 V, the spin polarization is defined as zero since there is no tunneling current regardless of gate voltage. (**c**) Spin-polarization of the tunneling current densities as functions of gate voltage for different bias voltages *V*_*b*_ = 50 and 100 mV. Insets: Energetic diagrams which describe the particular tunneling phenomena corresponding to the cases of the unity spin-polarizations, i.e., *P*_*j*_ = ±1. (**d**–**f**) Spin-resolved tunneling current densities as functions of the bias voltage for different gate voltages *V*_*G*_ = 0, −8, and 15 V, respectively. The results are calculated at *T* = 77 K.

As Fig. 2b exhibited, the tunneling current density is well spin-polarized for small amounts of bias voltage, |*V*_*b*_| < 0.1 V. Remarkably, within this bias voltage range, it is found that the current density can be fully spin-polarized according to gate voltage. This gate-tunable feature of the spin transport is led by the following mechanisms. When the equilibrium chemical potential *μ*_0_ is place in the mid-gap, the small bias voltage cannot lead to a sufficient amount of tunneling current densities for both spins. As bias voltage increases, spin-resolved current densities begin to flow. The spin-down current starts flowing slightly earlier than the spin-up current density because of their different electronic properties, i.e., the amount of band gaps and the position of the band edges. For large bias voltages, the current densities for both spins keep increasing with different increasing ratios associated to the spin-resolved Fermi velocities in SDOS. For *V*_*G*_ = 15 V, as plotted in Fig. 2a, an FMG layer is purely spin-down-polarized, and thus a pure spin-down current is generated by small bias voltages (see Fig. 2f). On the contrary, an application of *V*_*G *_= −8 V makes an FMG layer purely spin-up polarized, and the contribution to the tunneling current density is dominated by spin-up Dirac fermions for small bias voltages as shown in Fig. 2e. In other words, the spin-polarization of the tunneling current density can be switched according to the gate voltage, as shown in Fig. 2c. There is a very large contrast in the spin-polarization values around *V*_*G*_ = 0 V because the majority spin states near both band edges are opposite to each other (see Fig. 1c). Note that the spin transport phenomena are influenced by temperature, but this spin-switching effects are expected to be observed even at room temperature (see Supplementary Information). Besides, the spin-up contribution to the tunneling current is always dominant for the relatively larger bias voltages in Fig. 2d–f. This results from the fact that an FMG ends up spin-up-polarized as its Fermi level is tuned away from the band gap (see Fig. 1c).

### Tunneling current characteristics in FMG-FEI-FMG heterostructures

For FMG-NI-FMG heterostructures, the spin-resolved band structure of FMG is involved in the spin-polarized tunneling phenomena and the manipulation of the spin degree of freedom by means of an electric field via gate electrodes. The occurrence of pure spin-polarized current is attributed to the spin-resolved band gap of FMG, where only one spin states can be allowed near the band edges. This feature leads to purely spin-polarized FMG layers which can be utilized in the spintronic devices to explore a giant magnetoresistance (GMR). For typical ferromagnetic metal (FM)-NI-FM heterojunctions, electrical resistance strongly depends on how the FM configuration is set. While the electrical current flows well with the small resistance in the parallel configuration, a very large resistance is measured in the anti-parallel configuration. To achieve GMR, devices should be asymmetrically fabricated by using different kinds of FM materials, for which magnetization varies with respect to external magnetic fields. This means that controlling the magnetic fields is essential to change FM configuration. In addition, GER has already been introduced in a normal metal (NM)-FEI-NM heterojunction by using the asymmetric electrical response of a sandwiched FEI^37^. The key to GER is using electric fields instead of magnetic fields to achieve a giant change in electrical resistance, allowing greater convenience in generating distinct on/off signals in terms of technology. However, an asymmetric device has still been essential to make the potential barrier profile inside the FEI layer. Here, we present a way of achieving the emergence of GER by investigating vertical transport through FMG-FEI-FMG vertical heterostructures. Our device architecture not only has an ability to produce a giant resistance change by means of electric fields, but also does not require asymmetric fabrication.

The properties of an FEI are described by a simple model of the polarization density as a function of an external electric field,


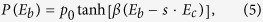


where *p*_0_ is the saturated polarization density, *β* is the characteristic coefficient with the physical dimension of inverse electric fields, 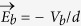 is an external electric field applied via bias voltage with the thickness of an FEI layer *d*, and *E*_*c*_ is the coercive field which is responsible for the hysterisis of the FEI. Here, the factor *s* = ±1 implies how the electric field varies, i.e. the forward or reverse sweep of electric fields (see Supplementary Information for the hysterisis of an FEI). The presence of the ferroelectricity in the tunnel barrier material is reflected two-fold. i) Carrier density on the FMG layers are influenced by the bound charge at FEI interfaces, 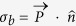. Accordingly, the spin-resolved Dirac cones are shifted by the amount of the charge imbalance between the FMGs. ii) The tunnel barrier profile is modulated by the bound charges in addition to the tunnel barrier caused by an external field. The former offers a rearrangement of the spin-resolved Dirac cones on FMG layers, and the latter accounts for the direction-dependent tunneling probability of Dirac fermions. Let us note that parameters used for the following calculations are *p*_0_ = 3.67 × 10^−3^ Cm^−2^, *E*_*c*_ = 4.55 × 10^6^ Vm^−1^, *β* = 6.6 × 10^−7^ mV^−1^, and *d* = 1 nm.

The shift of the spin-resolved Dirac cones is led by the following mechanism. For dual-gated devices, the carrier densities on FMG layers *n*_*T*_ and *n*_*B*_ are given as *n*_*T*_ = *n*_0_ − *δn*/2 and *n*_*B*_ = *n*_0_ + *δn*/2, where *δn* = *σ*_*b*_. The corresponding chemical potentials are determined by *n*_*T*_ and *n*_*B*_, i.e., 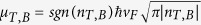, where *sgn*(*n*_*T*,*B*_) is the sign function. In equilibrium, the chemical potentials on the FMG layers should be arranged at the same Fermi energy to be consistent with equilibrium in the absence of bias voltage. Therefore, the Dirac cone on each FMG layer is shifted by *δμ*/2 = (*μ*_*T*_ − *μ*_*B*_)/2, respectively, i.e., 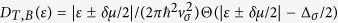 for the top and bottom FMGs. In fact, such Dirac cone shift coincides with a uniform electric field inside the tunneling layer *δμ*/*ed*. This FEI-induced electric field is reflected in the tunnling probability as below,


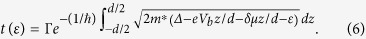


**Figure 3:**
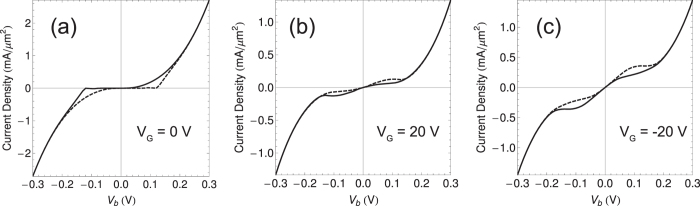
Total tunneling current densities versus bias voltage for different gate voltage in an FMG-FEI-FMG heterostructure. Solid and dashed lines represent the forward and reverse sweeps of bias voltage.

Figure 3 presents the vertical transport properties through FMG-FE-FMG heterostructures. Here, total current density is shown as a function of bias voltage, which is the sum of the spin-up and spin-down current densities. The tunneling current density clearly exhibits hysterisis behavior associated with FEI nature. For large bias voltage, the current density with the forward bias sweep is the same as that with the reverse bias sweep, resulting from the saturation of the polarization density. Total current density is resolved into spin-up and down current densities, and the spin-resolved feature is helpful in understanding the sweep-direction dependence (see Supplementary Information). Also, the current density behavior has considerable dependence on gate voltage.

### Giant electroresistance effects

**Figure 4:**
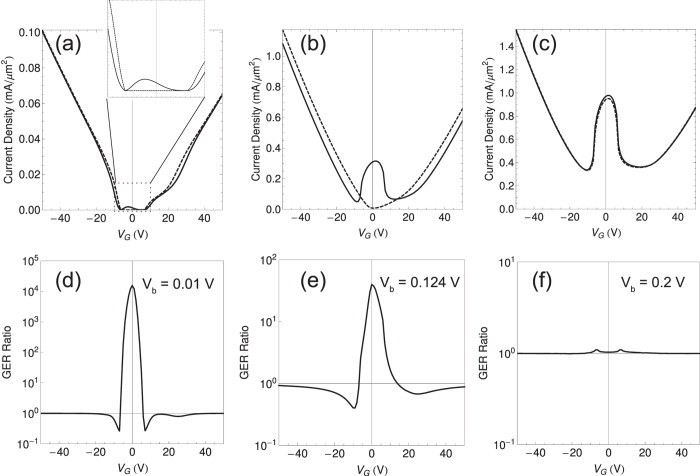
Giant electroresistance (GER) of FMG-FEI-FMG heterostructures. (**a**) Tunneling current densities versus gate voltage for *V*_*b*_ = ±0.01 V. Inset: Close-up of the current density plots for different bias voltage directions. (**b**,**c**) Tunneling current densities versus gate voltage for *V*_*b*_ = ±0.124 and ±0.2 V. (**d**–**f**) GER ratios as functions of gate voltage for different magnitudes of bias voltages, which correspond to (**a**,**b**,**c**), respectively. Solid and dashed lines represent the positive and the negative bias voltage. The results shown here are calculated at *T* = 77 K and for the forward sweep direction.

**Figure 5:**
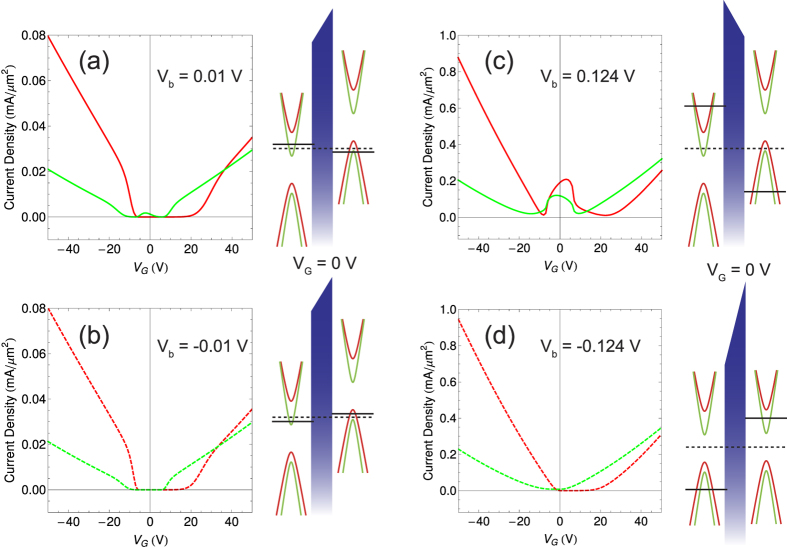
Spin-resolved vertical transport through FEI tunnel barriers for different bias voltage directions. (**a**,**b**) Plots of the tunnleing current densities attributed to different spins (Red: spin-up, Green: spin-down) as functions of gate voltage, for *V*_*b*_ = ±0.01 V, respectively. (**c**,**d**) Plots of the tunnleing current densities attributed to different spins (Red: spin-up, Green: spin-down) as functions of gate voltage, for *V*_*b*_ = ±0.124 V, respectively. Energetic diagrams next to each plot present the corresponding interpretations of the tunneling mechanism where the shift of the spin-resolved Dirac cones and the positions of the chemical potentials on the FMG layers. Dashed and solid black lines represent *μ*_0_ and *μ*_0_ ± *eV*_*b*_/2, respectively. Absolute values of the current density are shown for the both positive (solid lines) and negative (dashed lines) bias voltages.

Due to the hyeterisis feature, the current density values are expected to be asymmetric with respect to the bias voltage polarity, for a sweep direction of bias. Such an asymmetric response to bias voltage makes the current density 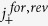 for positive bias voltage different from the current density 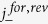 for the same magnitude of bias voltage with a negative sign, thereby resulting in a large ratio between them. Here, we define GER ratio as 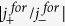 for the forward sweep direction and 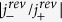 for the reverse sweep direction. As shown in Fig. 4d–f, the GER ratio converges to unity as gate voltage increase because of the fact that the FEI-induced shift of the Dirac cones cannot result in considerable differences in the FMG configuration. For convenience of comparison, the absolute values of the current densities are displayed. In general, the largest GER ratios are found around *V*_*G*_ = 0 V where chemical potentials reside near the band edges of FMGs. Further, for the very small bias *V*_*b*_ = 0.01 V, the tunneling current is allowed only by a positive bias, whereas it is strongly suppressed by a negative bias. Such a large GER ratio is led by the following mechanism. For very small bias voltages, the polarization density is almost unchanged from the saturated value, and the resulting Dirac cone shift makes one FMG layer purely spin-up polarized and the other FMG layer purely spin-down polarized, i.e., anti-parallel spin configuration of FMGs is derived. When a small bias is applied in the positive direction, the chemical potential on spin-up polarized FMG becomes lower and touches the lower spin-down band, while the chemical potential on spin-down polarized FMG becomes higher but still reside in the spin-down band only. On the other hand, the FMG configuration remains anti-parallel for *V*_*b*_ = −0.01 V, resulting in the suppression of vertical tunneling by Pauli blocking. Indeed, Fig. 5a shows that the current density for *V*_*b*_ = 0.01 V is influenced by the spin-down states only. Therefore, the GER ratio ~10^4^ originates from the bias-tunable spin-configuration of FMGs. Also, the GER ratio has dependence on temperature and deteriorates at higher temperatures (see Supplementary Information).

The effects of the FEI-induced Dirac cone shift are well interpreted in Fig. 5c,d. In this case, the applied bias is associated with the coercive fields, which make the polarization density of an FEI according to the sweep direction of bias voltage. For a forwardly sweeping bias, *V*_*b*_ = −0.124 V leads to zero polarization density, and *V*_*b*_ = +0.124 V makes the polarization density saturated. In other words, for *V*_*b*_ = −0.124 V, no shift is induced between two FMG layers, mimicking an FMG-NI-FMG heterostructure. Indeed, the tunneling current density exhibits behavior of typical vertical FETs where the tunneling current through an insulating layer is controlled by gate voltage. When the bias voltage is reversed to +0.124 V, the spin-resolved Dirac cones are shifted by the saturated polarization density of the FEI, and the tunneling current begins to flow even for zero gate voltage (see Fig. 5c,d). Due to the relatively large bias voltage, the energy window is wide enough to allow both spin-up and spin-down tunneling currents. In this case, the tunneling current density exhibits distinct behavior as gate voltage increases: the current density drops for specific gate voltage because the chemical potential of one FMG layer falls into a band gap, and then both chemical potentials reside in upper (or lower) bands, making the current density increases again as gate voltage increases.

## Summary and Discussion

Our results establish that an FMG vertical heterostructure is a potential platform for graphene-based spintronic devices. We demonstrated that tunneling current density can be spin-polarized through FMG-NI-FMG heterostructures, reaching up to unity. By using the spin-resolved band model of FMG, we revealed that vertical transport is accordingly spin-resolved. The spin transport through the FMG-NI-FMG heterostructure depends on the position of the equilibrium chemical potential, and its spin-polarization of the current density is tunable via gate voltage. In particular, in the vicinity of the FMG spin-resolved band gaps, there is a very drastic change in the spin-polarization between *P*_*j*_ = −1 and +1, leading to the gate-tunable spin-switching effects. This gate-tunable spin transport is attributed to the presence of the purely spin-polarized states in the FMG band model, which can be a good building block for GMR devices.

Accordingly, we demonstrated that the FMG heterostructure can be utilized to generate GER by replacing the NI with an FEI. While the FMG spin configuration is always parallel for FMG-NI-FMG heterostructures, having an FEI layer instead of an NI enables anti-parallel spin configuration for specific gate voltages due to the FEI-induced shift of the FMG bands. In the presence of the FEI layer between FMGs, the spin configuration is able to be manipulated by means of electric fields via bias voltage. With such a ‘magnetic-field free’ manipulation of the spin configuration, a giant resistance is achieved by controlling electric fields, contrary to GMR. Thus, GER has been proposed in this study through the investigation of FMG-FEI-FMG heterostructures.

As the proposed system has been theoretically studied in the absence of the external magnetic fields, there could be an interesting question about the direction of the magnetization of FMGs. In this study, the orientation of the FMG magnetization does not affect the results as two identical FMG layers were used for the proposed heterostructures. Although the magnetization is oriented along a preferable direction, the configuration of the magnetization should be parallel for FMG-NI-FMG heterostructures and either parallel or anti-parallel for FMG-FEI-FMG heterostructures according to the gate and bias voltages.

In conclusion, the gate tunability of the spin-switching effects and the GER implies that the operation of the spintronic devices proposed in this study does not require the use of magnetic fields. The prospect of the proposed system for practical spintronic applications can be also examined with studies of temperature dependence on the results.

## Additional Information

**How to cite this article**: Myoung, N. *et al.* Gate-Tunable Spin Transport and Giant Electroresistance in Ferromagnetic Graphene Vertical Heterostructures. *Sci. Rep.* **6**, 25253; doi: 10.1038/srep25253 (2016).

## Supplementary Material

Supplementary Information
