# Supplementary material for: Gate-Tunable Spin Transport and Giant Electroresistance in Ferromagnetic Graphene Vertical Heterostructures
Source: Sci Rep. 2016 Apr 29;6:25253. doi: 10.1038/srep25253 (PMC4850479; doi:10.1038/srep25253)
Supplement: Supplementary Information [file srep25253-s1.pdf]

Supplementary Information: Gate-Tunable Spin Transport and Giant Electroresistance in Ferromagnetic Graphene Vertical Heterostructures

Nojoon Myoung,[*,†,‡,§] Hee Chul Park,[‡,§] and Seung Joo Lee[*,¶]

†*Department of Material Science and Engineering, University of Ioannina, Ioannina 45110, Greece*

‡*Center for Theoretical Physics of Complex Systems, Institute for Basic Science, Daejeon 34051, Republic of Korea*

¶*Quantum-functional Semiconductor Research Center, Dongguk University, Seoul 100-715, Republic of Korea*

§*NM and HCP equally contribute to the present work*

E-mail: nmyoung@cc.uoi.gr; leesj@dongguk.edu

1. Ferroelectric insulator model and the spin-resolved tunneling current

Now, effects of the ferroelectricify are focussed on the rearrangement of the spin-resolved band structures of FMG as a way of controlling the spin configuration by means of electric fields. Figure 1 shows effects of the ferroelectricity on the vertical tunneling phenomena stage by stage. i) For $V_b = 0.1$ V, the spin-resolved Dirac cones on FMG layers are rearranged by the saturated polarization density, and both spin-up and down tunnelig currents are

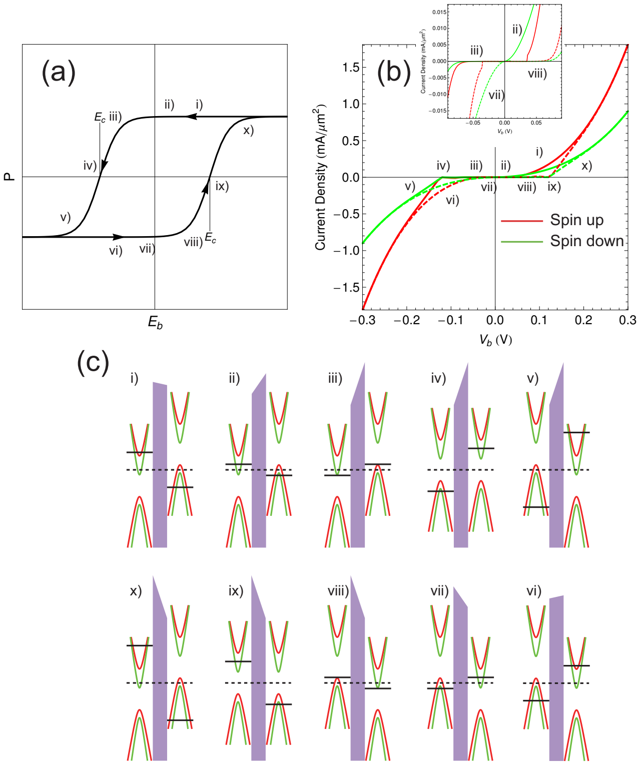

Figure 1: Effects on the ferroelectricity on the spin-resolved vertical transport. (a) Modeled hysterisis loop of the FEI in this study. Polarization density P is displayed as a function of an electric field induced via bias voltage. The arrows represent how the electric field varies, and the roman numerals inducate to various situations of tunneling phases corresponding to those in (b). (b) Spin-resolved tunneling current density as a function of bias voltage for $V_G = 0$. The solid and dashed lines represent the bias voltage sweep direction, i.e., the forward and reserve sweep, respectively. The inset shows a close-up of the spin-resolved current around zero bias voltage. (c) Schematic diagram of the tunneling phase corresponding to the romal numerals in (a,b). The solid and dashed horizontal lines denote μ_0 and $\mu_0 \pm eV_b/2$, respectively. Different shapes of the tunnel barriers herein imply the net electric field between FMG layers.

generated by the chemical potential difference between FMG layers.ii) When V_b decreases to a very small value, the polarization density is still saturated, so that the arrangement of the Dirac cones is not changed.The tunneling current is suppressed by the small bias voltage, but only spin-down states can contribute to the tunneling current by thermally excited Dirac fermions (note the position of the chemical potentials ii) diagram in Figure 1c). iii) At this state, each FMG layer is fully spin-polarized with opposite spin states, resulting into the suppression of the tunneling current. In this case, the anti-symmetric configuration of FMGs is achieved by electrical means. iv) As V_b increases toward the negative direction, an electric field reaches the coercive field E_c, resulting in a zero polarization density. Note that, at this stage, the shift of the spin-resolved Dirac cones vanishes due to the absence of the bound charge. v) By keep increasing bias voltage, the sign of the polarization density is reversed, and the tunneling current begin to increase according to the increase in the chemical potential difference between FMG layers. Here, the increasing behavior of the tunneling current for each spin is different from each other because of the aforementioned spin-resolved band model. From the stage vi) to x), V_b increases toward the positive direction from the stage v), and the tunneling phenomena follows the same mechanism described from the stage i) to v).

2. Temperature effects on the spin transport and the GER ratio

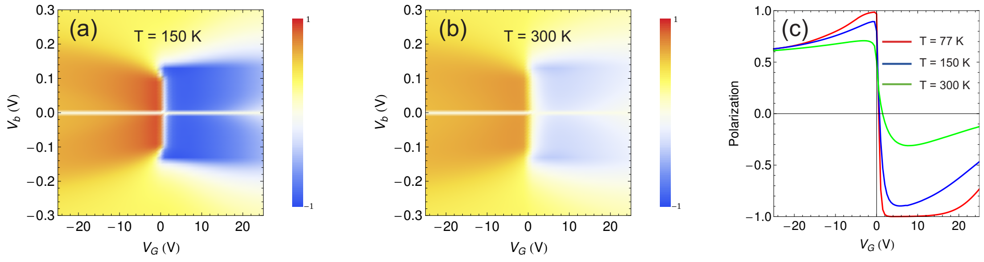

Figure 2: Temperature dependence of the spin-switching effect. (a,b) Color maps of the spin-polarization versus bias and gate voltages through FMG-NI-FMG heterostructures at $T = 150$ and 300 K, respectively. (c) Spin-polarization curves as functions of gate voltage at different temperatures for the given bias voltage $V_b = 0.05$ V.

Here, let us discuss temperature effects on the spin switching phenomena via gate voltage. discuss temperature effects on the spin transport phenomena through the FMG-NI-FMG heterostructure. The thermal distribution of Dirac fermions on each FMG layer is taken into account by the Fermi-Dirac distribution function in Eq. (2). Figure 2 clearly shows that the contrast in the spin-polarization becomes smaller at high temperatures. This is due to more carriers becoming thermally activated which results in reduced spin-polarization purity of the tunneling current density. However, even at room temperature, it is still expected to observe that the spin-polarization of the tunneling current density can be switched by adjusting gate voltage.

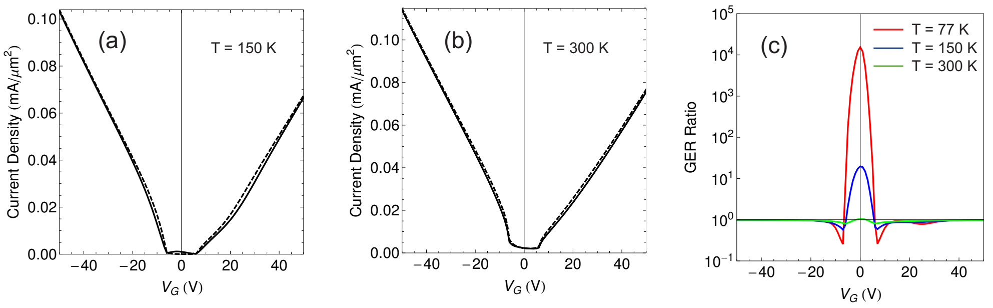

Figure 3: Temperature dependence on GER ratio. (a,b) Plots of the tunneling current density versus gate voltage for $V_b = 0.01$ V at $T = 150$ and 300 K. Solid and dashed lines represent the positive and negative bias voltages, respectively. (c) GER ratios as functions of gate voltage at different temperatures. The results shown here are calculated for the forward sweep direction.

Naturally, the GER ratio is also expected to be dependent on temperature. The results shown in Fig. 4 are obtained at the relatively low temperature of 77 K. As discussed, the spin-dependent vertical transport phenomena here are attributed to the spin-resolved band structures of FMG, and sufficient energetic resolution, which is at least smaller than the difference between the spin-resolved band gaps, is essential. Thus, it is anticipated that the GER ratio is reduced by temperature. Figure 3 shows the relationship between temperature dependence on GER ratio, and clearly agree with the anticipated reduction in the GER ratio with increasing temperature.
